# Editorial: Omics-based novel computational methods revealing microbe-disease associations

**DOI:** 10.3389/fcimb.2023.1305902

**Published:** 2023-10-26

**Authors:** Stephen S.-T. Yau, Qi Wu

**Affiliations:** ^1^ Department of Mathematical Sciences, Tsinghua University, Beijing, China; ^2^ Yanqi Lake Beijing Institute of Mathematical Sciences and Applications (BIMSA), Beijing, China; ^3^ Institute of Microbiology, Chinese Academy of Sciences (CAS), Beijing, China

**Keywords:** GWAS, drug resistance, microbiome, multifractal analysis, genome

It is with great pleasure that we announce the successful conclusion of our Research Topic, “*Omics-based Novel Computational Methods Revealing Microbe-Disease Associations*.” This dedicated platform has provided an exceptional opportunity to explore and advance the application of computational methods in the study of microbe-disease associations, shedding light on the intricacies of this field.

First and foremost, we extend our heartfelt gratitude to all the researchers, authors, and reviewers who have contributed to this Research Topic. Your dedication and professionalism have been pivotal to the success of this endeavor. We have received an impressive array of research contributions from around the world, covering a spectrum of microbe-disease associations, from fundamental science to clinical applications.

The central objective of this Research Topic has been to delve into the potential of novel computational methods within the “Omics” domain, encompassing genomics, transcriptomics, proteomics, and more. Our researchers have leveraged advanced computational techniques such as deep learning, machine learning, multifractal analysis and whole-genome association studies to unravel the complex relationships between microbes and various types of diseases. These methods not only offer new avenues of inquiry but also unprecedented opportunities for the future of disease prevention, diagnosis, and treatment.

A prominent research direction is the pattern of variant types in viruses. From the HIV-related study of Sun and Yau, we were able to see the ability of k-mer analysis in identification of different types of substitution mutations in virus genome sequences. This example reveals the connection between the mutation pattern and geographical distribution of HIV virus to address clinical challenges.

In addition to genomic research, the field of protein structure is also a fundamental component of our Research Topic. Lyu et al. investigated the predictions of interactions of tetrameric protein complexes. They found that, in combination with specific feature extraction methods, a Convolutional Neural Network (CNN) model was proven successful in predicting the interaction between chains of the tetramer protein complex. Furthermore, an ensemble approach utilizing the Support Vector Machine (SVM) has showcased remarkable efficacy in predicting the tetramer protein complex interface residue pairs. These advancements considerably expand our understanding and exploration of protein complex interaction mechanisms.

Within our Research Topic, certain studies have stood out. Chen et al. emphasized the exploration of drug resistance (or tolerance) in relation to microbial communities, particularly in clinical settings. Their investigations have not only provided profound insights into mechanisms of drug resistance/tolerance but also offered vital clues for the development of innovative therapeutic strategies.

Furthermore, our Research Topic has addressed the connection between microbial composition, function, and host health, along with the effective utilization of computational methods in studying these intricacies. Tan et al. described the importance of long chain non-coding RNAs (lncRNAs) in disease research, emphasizing that the study of lncRNA-disease associations is important for the diagnosis, treatment and prevention of diseases. Zhao et al. summarize classical mathematical models and deep learning methods in the general reconstruction step of cryo-electron tomography, while also discussing current limitations and prospects. Research in this field is poised to have a profound impact on our understanding of the interplay between microbes and disease. Xie et al. proposed a multifractal analysis method for metagenomic research, and they found that there is self-similarity between the chaos game representations (CGRs) of whole genome sequences of metagenomes, and the multifractal spectrum is an important feature of metagenomes. Based on this, they found that multifractal profiles are associated with infant development through an example analysis of the infant gut microbiome.

In closing, this Research Topic deeply explored the potential of novel computational methods in the field of “Omics”, and a variety of new research perspectives and tools have been brought to bear to unravel the complex relationship between microorganisms and various diseases. We express our heartfelt gratitude once again to all those who have contributed to this Research Topic. Your work will continue to guide the evolution of research in microbe-disease associations. We look forward to further collaborations and innovations in the quest to better comprehend the complexities of this field and make significant contributions to the improvement of human health.

## Author contributions

SY: Writing – original draft. QW: Writing – original draft.

